# Supplementary material for: Resolving the full spectrum of human genome variation using Linked-Reads
Source: Genome Res. 2019 Apr;29(4):635–45. doi: 10.1101/gr.234443.118 (PMC6442396; doi:10.1101/gr.234443.118)
Supplement: Supplemental Material [file supp_gr.234443.118_Supplemental_longranger-2.2.zip › longranger-2.2.tar.bz2!/lr2.2-public/lib/stan/cmdstan/stan/doc/api/html/fill_8hpp_source.html]

Stan: src/stan/math/matrix/fill.hpp Source File


|  |  |
| --- | --- |
|  | Stan  2.5.0  probability, sampling & optimization |


- Main Page
- Related Pages
- Namespaces
- Classes
- Files

- File List
- File Members

All Classes Namespaces Files Functions Variables Typedefs Enumerations Enumerator Friends Macros Pages

- src
- stan
- math
- matrix

fill.hpp

Go to the documentation of this file.

1 #ifndef STAN\_\_MATH\_\_MATRIX\_\_FILL\_HPP

2 #define STAN\_\_MATH\_\_MATRIX\_\_FILL\_HPP

3

4 #include <vector>

5 #include <stan/math/matrix/Eigen.hpp>

6

7 namespace stan {

8

9  namespace math {

10

21  template <typename T, typename S>

22  void fill(T& x, const S& y) {

23  x = y;

24  }

25

38  template <typename T, int R, int C, typename S>

39  void fill(Eigen::Matrix<T,R,C>& x, const S& y) {

40  x.fill(y);

41  }

42

54  template <typename T, typename S>

55  void fill(std::vector<T>& x, const S& y) {

56  for (size\_t i = 0; i < x.size(); ++i)

57  fill(x[i],y);

58  }

59

60

61

62  }

63 }

64 #endif

stan::math::fill

void fill(T &x, const S &y)

Fill the specified container with the specified value.

**Definition:** fill.hpp:22

Eigen.hpp


---

[ Stan Home Page ]

*© 2011–2014,
Stan Development Team.*
